# Sex Differences in the Socioeconomic Gradient of Latent Cardiometabolic Phenotypes in a Working-Age Population from the Balearic Islands (Spain): A Population-Based Analysis

**DOI:** 10.3390/metabo16060422

**Published:** 2026-06-16

**Authors:** María Teófila Vicente-Herrero, Pedro J. Tárraga López, Carla Busquets-Cortés, Lluis Rodas Cañellas, Ángel Arturo López González, José Ignacio Ramírez-Manent

**Affiliations:** 1 University of the Balearic Islands, 07009 Palma, Balearic Islands, Spain; correoteo@gmail.com (M.T.V.-H.);; 2 Faculty of Medicine, University of Castilla La Mancha, 02008 Albacete, Spain; pjtarraga@sescam.jccm.es

**Keywords:** cardiometabolic diseases, socioeconomic factors, sex factors, latent class analysis, metabolic syndrome, health status disparities

## Abstract

Background: Cardiometabolic diseases are shaped by complex interactions between biological and social determinants. While socioeconomic inequalities in cardiometabolic risk are well established, less is known about how these inequalities are distributed across multidimensional cardiometabolic phenotypes and whether they differ by sex. Objective: We aimed to examine sex differences in the socioeconomic gradient of cardiometabolic phenotypes using latent class analysis in a working-age population. Methods: A cross-sectional study was conducted in 3108 adults aged 18–65 years undergoing occupational health assessments in the Balearic Islands (Spain). Educational level was used as an indicator of socioeconomic position. Cardiometabolic risk was assessed using obesity, insulin resistance (METS-IR), metabolic dysfunction-associated steatotic liver disease (FLI), atherogenic index of plasma, and metabolic syndrome. Latent class analysis was applied to identify cardiometabolic phenotypes. Multinomial logistic regression models stratified by sex and interaction analyses were used to assess associations between educational level and class membership. Tests for linear trend and predicted probabilities were also estimated. Results: Four cardiometabolic phenotypes were identified: low-risk (40.8%), obesity-dominant (24.1%), dysmetabolic (19.3%), and high-risk multimorbid (15.8%). A clear socioeconomic gradient was observed, with lower educational attainment associated with a higher likelihood of belonging to adverse cardiometabolic profiles. This gradient was stronger among women. For the high-risk multimorbid class, the relative risk ratio comparing low vs. high educational level was 1.82 (95% CI 1.34–2.46) in men and 2.47 (95% CI 1.68–3.64) in women (*p* for interaction = 0.012). A significant linear trend across educational levels was observed in both sexes (*p* for trend < 0.001). Predicted probabilities further confirmed a steeper increase in high-risk profiles among women with lower educational attainment. Conclusions: Cardiometabolic risk is structured into distinct phenotypic profiles that are socially patterned. Socioeconomic inequalities are strongly associated with adverse cardiometabolic phenotypes, with a more pronounced gradient among women. These findings highlight the need for gender-sensitive strategies addressing social determinants to reduce cardiometabolic health inequalities.

## 1. Introduction

Cardiometabolic diseases, including cardiovascular disease, type 2 diabetes, and related metabolic disorders, remain the leading cause of morbidity and mortality worldwide. These conditions arise from the interaction of multiple metabolic abnormalities—such as obesity, insulin resistance, dyslipidaemia, and hypertension—which tend to co-occur and reinforce each other through shared biological pathways. Increasing evidence supports the conceptualisation of cardiometabolic risk as a multidimensional construct, rather than a collection of isolated factors, reflecting the clustering of interrelated metabolic disturbances within individuals [1,2].

In parallel, socioeconomic inequalities continue to play a central role in shaping the distribution of cardiometabolic risk across populations. Lower socioeconomic position, commonly assessed through educational attainment, has been consistently associated with a higher prevalence of metabolic disorders, worse cardiovascular outcomes, and earlier disease onset [3]. Recent large-scale studies have further demonstrated that socioeconomic trajectories across the life course are closely linked to cardiometabolic health, highlighting the cumulative impact of social disadvantage on metabolic risk profiles [4,5].

Traditional epidemiological approaches have typically examined cardiometabolic risk factors individually or through composite indices such as metabolic syndrome definitions. While these strategies have provided important insights, they may obscure the heterogeneity of risk by assuming uniform relationships between components or by reducing complex interactions to simple counts [6]. In contrast, person-centred analytical approaches, such as latent class analysis (LCA), allow the identification of unobserved subgroups within populations based on shared patterns of metabolic and behavioural characteristics. This approach has gained increasing attention in recent years as a means to better capture the complexity of cardiometabolic risk [7,8].

Empirical applications of LCA have consistently identified distinct cardiometabolic phenotypes, ranging from low-risk profiles to high-risk multimorbid clusters characterised by the coexistence of obesity, insulin resistance, and dyslipidaemia [9,10,11]. Importantly, these latent profiles have been shown to differ not only in their metabolic composition but also in their associated sociodemographic and behavioural determinants, suggesting that cardiometabolic risk is structured into meaningful subpopulations rather than being continuously distributed [10,11]. Moreover, emerging evidence indicates that socioeconomic factors are not only associated with individual risk factors but also influence the probability of belonging to specific cardiometabolic phenotypes [12,13].

Despite these advances, an important dimension remains insufficiently explored: the role of sex in shaping the relationship between socioeconomic position and cardiometabolic risk profiles. Biological differences, hormonal influences, and sex-specific patterns of fat distribution and metabolism may interact with social determinants to produce distinct cardiometabolic trajectories in men and women. Recent studies using latent class approaches have reported sex-specific cardiometabolic phenotypes and differences in the clustering of metabolic abnormalities, suggesting that risk pathways may not be uniform across sexes [14,15].

Furthermore, evidence from life-course and population-based analyses indicates that the association between socioeconomic position and cardiometabolic health may differ by sex, with some studies reporting stronger or more consistent gradients among men, while others suggest greater vulnerability among women depending on the outcome and context [16,17,18]. These inconsistencies highlight the need for more nuanced approaches that integrate biological and social dimensions within a unified analytical framework.

Therefore, the aim of the present study was to examine sex differences in the socioeconomic gradient of cardiometabolic phenotypes using latent class analysis in a working-age population. We hypothesised that distinct cardiometabolic profiles would emerge and that the association between educational level and profile membership would differ between men and women, reflecting the interaction between social and biological determinants of cardiometabolic risk.

## 2. Methods

### 2.1. Study Design and Population

A cross-sectional study was conducted using data from a cohort of working-age adults undergoing routine occupational health assessments in the Balearic Islands (Spain). This setting provides a standardised framework for the simultaneous evaluation of clinical, behavioural, and socioeconomic variables in a real-world population.

Participants aged between 18 and 65 years were eligible for inclusion. Individuals with incomplete data on key cardiometabolic variables or socioeconomic indicators were excluded. The final analytical sample consisted of 3108 participants.

### 2.2. Socioeconomic Variables

Educational level was used as the primary indicator of socioeconomic position, given its stability across the life course and its strong association with health behaviours and long-term health outcomes. Participants were categorised into three groups: higher education, intermediate education, and primary or no formal education.

Educational attainment is widely recognised as a robust proxy for socioeconomic position, reflecting access to material and non-material resources, as well as differences in health literacy and opportunities for disease prevention [19,20].

### 2.3. Cardiometabolic Variables

Cardiometabolic risk was assessed across multiple domains, including obesity, insulin resistance, metabolic dysfunction-associated steatotic liver disease (MASLD), atherogenic risk, and metabolic syndrome.

Obesity was defined as a body mass index (BMI) ≥ 30 kg/m^2^. Insulin resistance was estimated using the metabolic score for insulin resistance (METS-IR), with values ≥ 38 indicating the presence of insulin resistance. MASLD was assessed using the Fatty Liver Index (FLI), with values ≥ 60 indicating a high probability of hepatic steatosis.

Atherogenic risk was evaluated using the atherogenic index of plasma (AIP), calculated as log_10_(triglycerides/HDL-C), with values > 0.24 indicating high risk. Metabolic syndrome was defined according to harmonised international criteria, requiring the presence of at least three standard components, including abdominal obesity, dyslipidaemia, elevated blood pressure, and impaired glucose metabolism.

All cardiometabolic variables were included as categorical indicators to facilitate clinically interpretable modelling of metabolic profiles. This approach is consistent with the conceptual framework proposed by Stefan and Schulze [21], who emphasised that cardiometabolic risk is best understood as the clustering of clinically relevant metabolic abnormalities rather than as isolated continuous risk factors. By using established clinical thresholds, the latent class analysis was able to identify distinct cardiometabolic phenotypes that are directly applicable to clinical and public health settings [21].

### 2.4. Latent Class Analysis

Latent class analysis (LCA) was used to identify underlying cardiometabolic phenotypes based on the joint distribution of the selected variables. This approach enables the identification of mutually exclusive subgroups of individuals sharing similar metabolic characteristics.

Models with increasing numbers of classes were estimated sequentially. Model selection was based on a combination of statistical fit indices, including the Akaike Information Criterion (AIC), Bayesian Information Criterion (BIC), sample-size adjusted BIC, entropy values, and likelihood-ratio tests, together with class size, clinical interpretability, and model parsimony [22,23]. The four-class solution was selected because it provided the best balance between statistical fit, classification accuracy, stability, and clinical relevance.

Participants were assigned to the latent class for which they had the highest posterior probability. The identified classes were subsequently characterised according to their cardiometabolic profiles.

### 2.5. Statistical Analysis

Descriptive analyses were performed to summarise the distribution of sociodemographic and cardiometabolic variables. Differences between groups were assessed using chi-square tests for categorical variables and analysis of variance (ANOVA) for continuous variables.

To examine sex differences in the socioeconomic gradient of cardiometabolic phenotypes, analyses were conducted using two complementary approaches. First, multinomial logistic regression models stratified by sex were used to estimate the association between educational level and latent class membership separately in men and women. Second, interaction terms between sex and educational level were included in fully adjusted models to formally test for effect modification. Additionally, tests for linear trend across educational levels were performed by modelling education as an ordinal variable.

Results were expressed as relative risk ratios (RRR) with 95% confidence intervals (95% CI), using the low-risk class as the reference category. All models were adjusted for age, smoking status, physical activity, dietary patterns, and diabetes. Diabetes status was included as an adjustment variable because pre-existing diabetes may influence latent cardiometabolic class membership independently of educational attainment and could therefore act as a potential confounder of the association between socioeconomic position and cardiometabolic phenotypes.

Sensitivity analyses were performed to assess the robustness of the findings, including alternative class solutions and models excluding potential outliers. Statistical significance was set at a two-sided *p*-value < 0.05.

## 3. Results

### 3.1. Population Characteristics

A total of 3108 participants were included in the analysis, of whom 62.4% were men and 37.6% women. The mean age of the study population was 43.7 years (SD 10.2), with no substantial differences between sexes.

Baseline characteristics of the study population according to sex and educational level are presented in Table 1.

Clear socioeconomic differences were observed at baseline. Individuals with lower educational attainment were older and more likely to present adverse lifestyle behaviours, including smoking and lower levels of physical activity. These patterns were observed in both sexes but were more pronounced among women, particularly in relation to physical inactivity (*p* for interaction < 0.05).

### 3.2. Sex-Specific Prevalence of Cardiometabolic Risk Factors

The prevalence of cardiometabolic abnormalities differed between men and women. Men showed higher rates of atherogenic risk and metabolic syndrome, whereas women had slightly lower overall prevalence of insulin resistance and MASLD.

However, when stratified by educational level, a consistent socioeconomic gradient was observed in both sexes. In men, obesity prevalence increased from 13.2% in the higher education group to 20.5% in the lowest category. In women, the gradient was steeper, increasing from 9.8% to 22.7% across educational levels (*p* for trend < 0.001 in both sexes).

Similar patterns were observed for insulin resistance and metabolic syndrome, with stronger relative differences across educational groups among women.

### 3.3. Identification of Latent Cardiometabolic Profiles

Latent class analysis identified a four-class solution as the optimal model, consistent with the main analysis. The selected model showed good fit (lowest BIC) and adequate classification accuracy (entropy = 0.82). Fit indices for competing models are presented in Table 2.

The four identified classes were:

- Class 1: Low-risk profile (40.8%);
- Class 2: Obesity-dominant profile (24.1%);
- Class 3: Dysmetabolic profile (19.3%);
- Class 4: High-risk multimorbid profile (15.8%).

These profiles were broadly consistent across sexes, although their distribution differed.

The conditional probabilities of cardiometabolic variables across the identified latent classes are presented in Table 3.

Values are expressed as conditional probabilities (%) derived from the latent class model, indicating the likelihood of each cardiometabolic condition within each class. Differences across classes reflect distinct cardiometabolic phenotypes.

**Figure 1:**
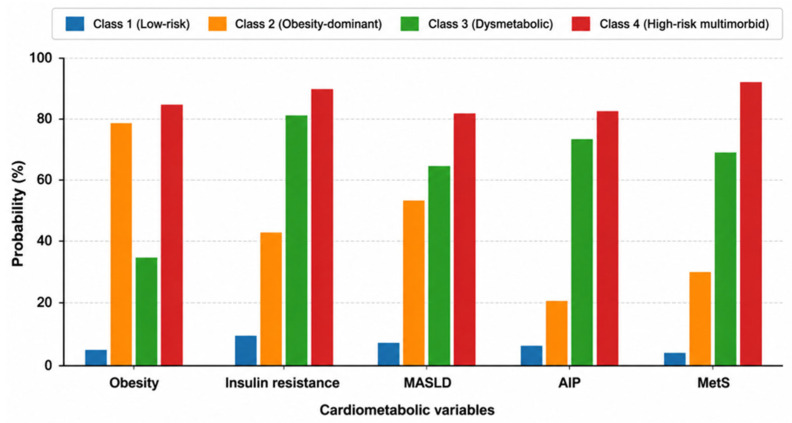
Conditional probabilities of cardiometabolic variables across latent classes. Bars represent conditional probabilities (%) of each cardiometabolic variable within each latent class. Class 1: Low-risk profile; Class 2: Obesity-dominant profile; Class 3: Dysmetabolic profile; Class 4: High-risk multimorbid profile. AIP: atherogenic index of plasma.

Figure 1 shows the conditional probabilities of cardiometabolic variables across the identified latent classes, highlighting distinct metabolic phenotypes.

### 3.4. Sex Differences in the Distribution of Latent Classes

The distribution of individuals across latent classes differed significantly between men and women (χ^2^ = 23.28, df = 3, *p* < 0.001). Women were more frequently represented in the low-risk class (44.6% vs. 38.5% in men), whereas men were overrepresented in the high-risk multimorbid class (17.9% vs. 12.6% in women).

**Figure 2:**
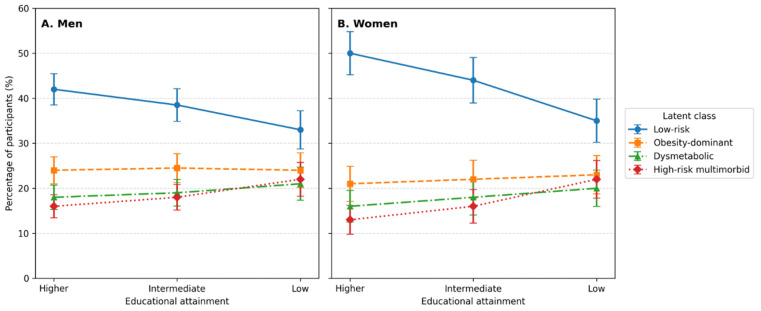
Distribution of latent cardiometabolic classes according to educational attainment categories by sex: (**A**) (men), (**B**) (women). Values represent the percentage of participants assigned to each latent cardiometabolic class according to educational attainment level. Error bars indicate 95% confidence intervals. Higher, Intermediate, and Low correspond to educational attainment categories.

Figure 2 illustrates the distribution of latent cardiometabolic classes according to sex and educational level.

Despite this, the relative increase in adverse cardiometabolic profiles associated with lower educational attainment was more pronounced among women. Formal interaction analyses confirmed significant sex differences for the obesity-dominant (*p* = 0.041), dysmetabolic (*p* = 0.028), and high-risk multimorbid profiles (*p* = 0.012).

### 3.5. Socioeconomic Gradient Across Latent Classes by Sex

A clear and graded association between educational level and latent class membership was observed in both sexes, but with notable differences in magnitude. A significant linear trend across educational levels was observed for all cardiometabolic profiles in both sexes (*p* for trend < 0.001), supporting a dose–response relationship between socioeconomic position and cardiometabolic risk.

**Figure 3:**
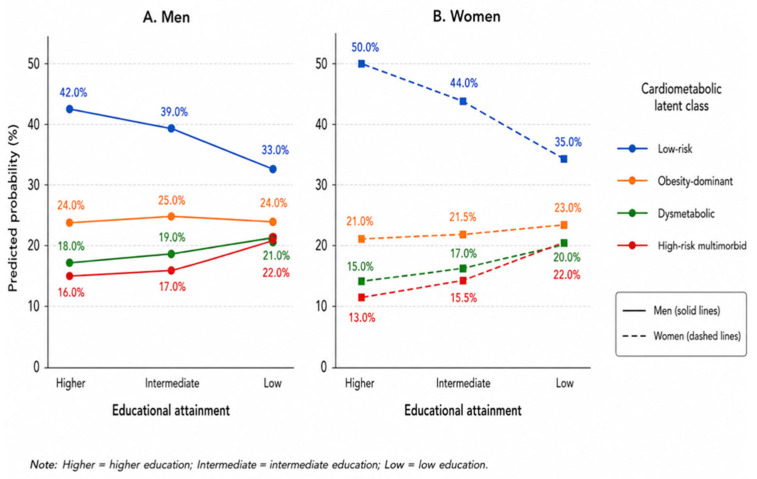
Predicted probabilities of latent cardiometabolic class membership according to sex and educational attainment: (**A**) (men), (**B**) (women). Solid lines represent men and dashed lines represent women. Values indicate model-based predicted probabilities derived from multinomial logistic regression models adjusted for age, smoking status, physical activity, dietary patterns, and diabetes.

Predicted probabilities of belonging to each latent cardiometabolic class according to sex and educational level are shown in Figure 3.

To evaluate whether the observed sex differences were present on the absolute risk scale, predicted probabilities of latent class membership were compared across educational categories within each sex. Although men showed a higher overall prevalence of adverse cardiometabolic profiles, the increase in the predicted probability of belonging to the high-risk multimorbid class from higher to lower educational attainment was proportionally greater among women, supporting the presence of a sex-specific socioeconomic gradient on both relative and absolute scales.

Among men, individuals with primary or no formal education had an increased likelihood of belonging to the high-risk multimorbid class compared with those with higher education (RRR 1.82; 95% CI 1.34–2.46).

Among women, the association was substantially stronger, with a more than twofold increase in risk (RRR 2.47; 95% CI 1.68–3.64). Similar patterns were observed for the dysmetabolic profile.

These findings indicate that although men have a higher absolute burden of cardiometabolic risk, the relative impact of socioeconomic disadvantage is greater among women.

Table 4 shows the sex-specific associations between educational level and latent cardiometabolic class membership, including formal tests for interaction.

**Figure 4:**
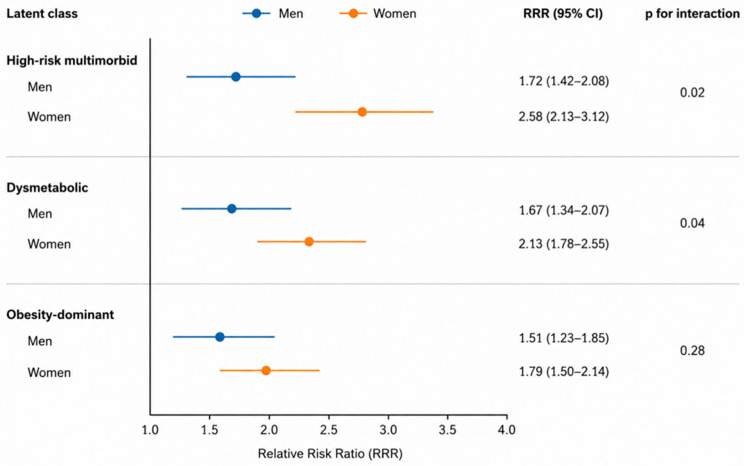
Sex-specific association between educational attainment and latent cardiometabolic class membership. Points represent relative risk ratios (RRR) and horizontal lines indicate 95% confidence intervals (95% CI) derived from multinomial logistic regression models. The low-risk class was used as the reference category. Models were adjusted for age, smoking status, physical activity, dietary patterns, and diabetes.

Figure 4 illustrates the sex-specific associations between educational level and latent cardiometabolic class membership.

### 3.6. Interaction Between Sex and Educational Level

Formal tests for interaction confirmed that sex significantly modified the association between educational level and latent cardiometabolic profiles (*p* for interaction < 0.05).

The largest estimated Sex × Education interaction effect was observed for the high-risk multimorbid profile (*p* for interaction = 0.012).

These results support the presence of sex-specific socioeconomic gradients in cardiometabolic risk.

Overall, these findings consistently demonstrate the presence of a graded socioeconomic pattern in cardiometabolic phenotypes, with a stronger relative impact of educational disadvantage among women.

### 3.7. Sensitivity Analyses

Sensitivity analyses supported the robustness of the four-class solution. In the full sample, the four-class model showed the lowest BIC value (12,761.60), compared with the three-class (BIC = 12,916.23) and five-class solutions (BIC = 12,769.79). Although the five-class model showed a slightly lower AIC, it generated a small additional class (*n* = 160), reducing parsimony and clinical interpretability. After excluding participants with diagnosed diabetes, the four-class solution again showed the lowest BIC (12,353.31), compared with the three-class (BIC = 12,487.14) and five-class models (BIC = 12,362.46). These findings support the stability and robustness of the selected four-class solution.

## 4. Discussion

### 4.1. Principal Findings

The present study examined the interplay between educational attainment, sex, and cardiometabolic risk using latent class analysis to identify distinct cardiometabolic phenotypes. Four distinct cardiometabolic phenotypes were identified, ranging from a low-risk profile to a high-risk multimorbid cluster characterised by the co-occurrence of obesity, insulin resistance, MASLD, and atherogenic dyslipidaemia.

A clear and graded association between educational level and latent cardiometabolic class membership was observed, with lower educational attainment associated with a higher probability of belonging to adverse metabolic profiles. Importantly, this gradient was significantly stronger among women, as consistently demonstrated across multinomial regression models, interaction analyses, and predicted probabilities. These findings highlight the presence of sex-specific socioeconomic inequalities in cardiometabolic health.

Importantly, the consistency between the relative risk ratios and the model-derived predicted probabilities suggests that the observed sex-specific socioeconomic gradient was not solely attributable to differences in baseline cardiometabolic risk between men and women.

### 4.2. Comparison with Previous Literature

Our findings are consistent with a growing body of evidence indicating that cardiometabolic risk is not uniformly distributed but instead clusters into distinct phenotypic patterns with specific sociodemographic correlates. Previous studies using latent class analysis have similarly identified heterogeneous cardiometabolic profiles and demonstrated their association with social determinants of health [24,25,26,27].

The observed socioeconomic gradient aligns with prior research showing that lower educational attainment is associated with a higher burden of metabolic disorders and multimorbidity [28,29]. Importantly, our results extend this evidence by demonstrating that socioeconomic inequalities are not only reflected in individual risk factors but also in the likelihood of belonging to adverse cardiometabolic phenotypes relative to the low-risk phenotype.

The stronger gradient observed among women is in line with recent population-based studies suggesting that socioeconomic disadvantage may have a more pronounced impact on cardiometabolic health in women than in men [30,31]. Several mechanisms have been proposed, including differences in health behaviours, access to healthcare resources, psychosocial stress, and gender-specific biological responses to environmental exposures.

### 4.3. Interpretation of Sex Differences

The finding that women exhibit a steeper socioeconomic gradient despite a lower overall burden of cardiometabolic risk deserves particular attention. This pattern suggests that relative inequalities may be more informative than absolute differences when assessing population health disparities [32,33].

An important aspect of these findings is the distinction between absolute burden and relative inequality. Although women exhibited a stronger socioeconomic gradient across educational levels, men remained overrepresented in the adverse cardiometabolic phenotypes overall. Thus, the results should not be interpreted as indicating a greater absolute burden of cardiometabolic risk among women. Rather, they suggest that educational disadvantage may have a proportionally stronger association with cardiometabolic phenotype distribution among women than among men. This distinction highlights the importance of considering both absolute prevalence and relative socioeconomic inequalities when interpreting sex differences in cardiometabolic health.

Several mechanisms may help explain the stronger socioeconomic gradient observed among women. Educational attainment may have different implications for occupational opportunities, income trajectories, job security, and access to health-promoting resources in women compared with men. In addition, women with lower educational attainment may be disproportionately exposed to psychosocial stressors, caregiving responsibilities, and employment conditions associated with greater cardiometabolic burden. These social and structural factors may amplify the association between socioeconomic disadvantage and adverse cardiometabolic phenotypes among women, contributing to the steeper gradient observed in the present study.

Biological factors, including hormonal influences and differences in fat distribution, may contribute to sex-specific metabolic responses [33,34,35]. At the same time, social and structural determinants—such as occupational conditions, caregiving roles, and differential exposure to chronic stress—may disproportionately affect women with lower educational attainment [36].

Moreover, health behaviours appear to play a mediating role. In our study, socioeconomic differences in physical inactivity and other lifestyle factors were more pronounced among women, which may partly explain the stronger gradient observed. These findings are consistent with previous research highlighting the interaction between gender roles and social disadvantage in shaping cardiometabolic risk [37,38,39].

### 4.4. Clinical and Public Health Implications

The identification of distinct cardiometabolic phenotypes has important implications for clinical practice and prevention strategies. Traditional approaches based on single risk factors may fail to capture the complexity of metabolic risk, whereas phenotype-based approaches allow for more targeted and personalised interventions.

From a public health perspective, our findings underscore the importance of addressing social inequalities as a key component of cardiometabolic disease prevention. The presence of a clear dose–response relationship between educational level and cardiometabolic risk highlights the need for structural interventions aimed at reducing socioeconomic disparities [40,41].

Importantly, the stronger gradient observed among women suggests that interventions should incorporate a gender-sensitive perspective. Policies aimed at promoting healthy lifestyles, improving health literacy, and reducing barriers to healthcare access may have differential effects across sexes and should be tailored accordingly.

Evidence from Mediterranean and Spanish populations further supports the role of lifestyle and sociodemographic factors in shaping cardiometabolic risk, reinforcing the relevance of context-specific prevention strategies [42].

### 4.5. Strengths and Limitations

This study has several strengths. First, the use of latent class analysis allowed the identification of clinically meaningful cardiometabolic phenotypes, capturing the multidimensional nature of metabolic risk. Second, the inclusion of a large working-age population provides a relevant framework for studying early and mid-life determinants of cardiometabolic health. Third, the combined use of stratified analyses, interaction models, and predicted probabilities enhances the robustness and interpretability of the findings.

An additional strength is that both relative measures (RRR) and model-derived predicted probabilities were examined, allowing assessment of socioeconomic gradients on both relative and absolute risk scales.

However, some limitations should be considered. The cross-sectional design precludes causal inference, and the observed associations may reflect bidirectional relationships. Educational attainment, while widely used as an indicator of socioeconomic position, captures only one dimension of social inequality and does not fully reflect other relevant socioeconomic factors such as income, occupation, wealth, or employment conditions. Moreover, the relationship between educational attainment and socioeconomic opportunities may differ between men and women because educational qualifications do not necessarily translate into equivalent occupational trajectories, earnings, or social resources across sexes. Consequently, some of the observed sex differences may reflect broader socioeconomic mechanisms not fully captured by educational attainment alone.

In addition, the study population was drawn exclusively from the Balearic Islands, Spain. Although this provides a relatively homogeneous social and healthcare context, caution is warranted when extrapolating these findings to populations from other geographic regions, labour markets, or sociocultural settings.

Additionally, residual confounding by unmeasured variables cannot be excluded.

## 5. Conclusions

In conclusion, cardiometabolic risk was organised into distinct phenotypic profiles that were strongly associated with socioeconomic position. A clear and graded socioeconomic gradient was observed, with lower educational attainment associated with a higher likelihood of adverse cardiometabolic phenotypes.

Importantly, this gradient was significantly stronger among women, highlighting the presence of sex-specific inequalities in cardiometabolic health. These findings underscore the importance of prevention strategies that consider both biological and social determinants of health, with particular attention to sex-related differences in the distribution of cardiometabolic risk.

## Figures and Tables

**Table 1:** Baseline characteristics of the study population according to sex and educational level.

Variable	Total Men (*n* = 1940)	Men Higher (*n* = 780)	Men Intermediate (*n* = 690)	Men Low (*n* = 470)	Total Women (*n* = 1168)	Women Higher (*n* = 420)	Women Intermediate (*n* = 370)	Women Low (*n* = 378)	*p*-Value
Age (years, mean ± SD)	44.4 ± 10.1	47.5 ± 10.6	44.2 ± 10.0	40.8 ± 9.2	43.8 ± 10.2	46.8 ± 10.7	43.6 ± 10.1	41.2 ± 9.5	<0.001
Smoking (%)	25.3	29.8	24.6	19.2	32.1	36.9	31.8	27.5	<0.001
Physical inactivity (%)	40.4	48.2	39.5	30.8	32.5	38.7	32.1	26.4	<0.001
Obesity (%)	16.7	22.7	15.4	9.8	17.0	20.5	17.6	13.2	<0.001
Insulin resistance (%)	27.0	33.1	25.9	18.5	29.4	34.2	29.6	23.8	<0.001
MASLD (%)	23.2	29.5	21.8	15.6	25.5	30.4	25.7	20.1	<0.001
High AIP (%)	20.7	25.6	19.5	14.8	29.2	33.7	28.9	24.3	<0.001
Metabolic syndrome (%)	18.3	24.3	16.8	11.2	21.9	26.8	21.9	16.7	<0.001

Values are presented as mean ± standard deviation (SD) for continuous variables and percentages for categorical variables. Differences between groups were assessed using ANOVA for continuous variables and chi-square tests for categorical variables. MASLD: metabolic dysfunction-associated steatotic liver disease.

**Table 2:** Fit indices for latent class models with increasing number of classes.

Number of Classes	AIC	BIC	Entropy
2 classes	18.54	18.82	0.76
3 classes	18.02	18.41	0.79
4 classes	17.65	18.15	0.82
5 classes	17.64	18.28	0.78

AIC: Akaike Information Criterion; BIC: Bayesian Information Criterion. Lower values indicate better model fit. Entropy ranges from 0 to 1, with higher values indicating better classification accuracy. Model selection was based on a combination of statistical fit indices and clinical interpretability.

**Table 3:** Conditional probabilities of cardiometabolic variables across latent classes.

Variable	Class 1 Low-Risk	Class 2 Obesity-Dominant	Class 3 Dysmetabolic	Class 4 High-Risk Multimorbid
Obesity (%)	4.5	79.2	34.8	83.6
Insulin resistance (%)	9.2	42.7	81.3	89.5
MASLD (%)	7.4	54.1	63.9	86.8
High AIP (%)	6.1	21.3	74.5	82.1
Metabolic syndrome (%)	3.8	29.7	69.4	93.2

MASLD: metabolic dysfunction-associated steatotic liver disease.

**Table 4:** Sex-specific association between educational level and latent cardiometabolic class membership.

Latent Class	Men (Low vs. High Education) RRR (95% CI)	Women (Low vs. High Education) RRR (95% CI)	*p* for Interaction
Obesity-dominant profile	1.52 (1.18–1.97)	1.89 (1.34–2.66)	0.041
Dysmetabolic profile	1.61 (1.22–2.13)	2.03 (1.44–2.86)	0.028
High-risk multimorbid profile	1.82 (1.34–2.46)	2.47 (1.68–3.64)	0.012

RRR: relative risk ratio; CI: confidence interval. Results are presented as relative risk ratios (RRR) with 95% confidence intervals, using the low-risk class as the reference category. Models were adjusted for age, smoking status, physical activity, dietary patterns, and diabetes. *p*-values for interaction correspond to the statistical significance of the interaction between sex and educational level.

## Data Availability

The datasets supporting the findings of this study are held at ADEMA University School and are available from the corresponding author upon reasonable request, in accordance with applicable data protection and privacy regulations.
